# Evaluation of the impact of a psycho-educational intervention on knowledge levels and psychological outcomes for people diagnosed with Schizophrenia and their caregivers in Jordan: a randomized controlled trial

**DOI:** 10.1186/1471-244X-14-17

**Published:** 2014-01-22

**Authors:** Abd Alhadi Hasan, Patrick Callaghan, Joanne S Lymn

**Affiliations:** 1 School of Health Sciences, Queen’s Medical Centre, University of Nottingham, Nottingham NG7 2UH, UK

**Keywords:** Schizophrenia, Schizoaffective, Primary caregivers, Trial, Psycho-education

## Abstract

**Background:**

Schizophrenia is one of the most serious forms of mental illness among people being treated in psychiatric clinics in developing and developed countries. Providing care for people diagnosed with schizophrenia can be stressful for their caregivers. Psycho-educational interventions may improve patients’ and primary caregivers’ knowledge of schizophrenia and impact positively on patients’ physical and psychological outcomes and primary caregivers’ burden of care and quality of life. Studies thus far have shown that these interventions may improve patients’ and caregivers’ outcomes, but the quality of included randomized controlled trials (RCTs) is poor and it is difficult to draw firm conclusions as to the effectiveness of such interventions on patients and primary caregivers’ outcomes, hence the current study.

**Methods/Design:**

A randomized controlled trial in four outpatient mental health clinics in Jordan comparing psycho-educational interventions in the form of six booklets every fortnight, with treatment as usual in people diagnosed with schizophrenia and their primary caregivers. The primary outcome for participants is knowledge of Schizophrenia; secondary outcomes for patients are positive and negative symptoms of schizophrenia and relapse rate, while secondary outcomes for primary caregivers are burden of care and quality of life. All measures are assessed at baseline, immediately post-intervention and at three months follow-up.

**Discussion:**

This randomized control trial, conducted in Jordan among people living with schizophrenia and their primary caregivers, will assess the effect of psycho-educational interventions on knowledge of Schizophrenia, patients’ positive and negative symptoms and quality of life, and caregivers’ burden of care.

**Trial registration:**

Current Controlled Trials ISRCTN78084871

## Background

Schizophrenia is one of the most common and serious forms of mental illness and is often chronic, recurrent, disabling and debilitating [1].

While studies have reported that the main cause of schizophrenia is unknown, a widely accepted model is the stress vulnerability hypothesis, which proposes that the interaction between biological vulnerability and socio-environmental stressors, including social stressors, have a significant role in the presentation and illness course [2]. This model suggests that schizophrenia is caused by an imbalance in biological or psychological systems. Imbalance in biological systems is considered the main precipitating cause for schizophrenia, and it includes genetics, head injury and viral infection. The impact of schizophrenia is commonly mitigated by taking medication and abstaining from alcohol. The psychological system is concerned with stress; stress is caused by events that challenge people and compel them to adapt themselves in order to function as ‘normal’. However, people who struggle to adapt to stressful life events (e.g. bereavement, loss of job) often report poorer disease symptoms [3].

Previous studies have estimated that schizophrenia affects around 1.1% of the adult population worldwide, which equates to around 51 million people. Commonly, people are diagnosed with schizophrenia before the age of 25 years [4].

The health system in Jordan has three sectors: Ministry of Health (MoH), private and military. The MoH provides healthcare to the majority of the Jordanian population [5].

In Jordan, a recent report [5] has shown that 305 individuals per 100,000 of the population have been diagnosed with mental illness, 50% of whom are diagnosed with schizophrenia, making this a significant healthcare issue.

Family interventions described in previous randomized controlled trials (RCTs) [6-8] are psycho-educational in nature and seek to improve patients and primary caregivers’ knowledge of schizophrenia and change patients and primary caregivers’ behaviour by improving their knowledge about expected ‘maladaptive’ behaviour, disease symptoms and how can they deal with these issues using psycho-educational interventions [9]. Whilst the content psycho-educational intervention varies between studies, there are common factors among most studies [7,10-12] including; general information about schizophrenia, symptoms, medication management, problem-solving strategies and communication skills for patients and primary caregivers. Psycho-educational interventions are usually delivered by psychiatrists [13]. mental health nurses [6,7,13] and social workers [14]. The average duration of sessions varies among studies from 60 to 120 minutes [7,10,15-17], The methods of delivering psycho-educational interventions in studies for people diagnosed with Schizophrenic patients and primary caregivers include lectures [7,10,18-20], face to face methods supported with a printed booklet [10,13] and online education [20].

Studies which adopted an online method of delivering psycho-educational interventions to participants revealed a substantial improvement in patients diagnosed with schizophrenia and caregivers’ knowledge levels and patients’ symptoms [8], stress and social support levels [20]. Additionally, delivering psycho-educational interventions with minimal interaction such as printed booklets and online provision has shown a similar effect on participants’ outcomes [20]. A recent meta-analysis [21] of RCTs that delivered psycho-educational interventions online, by email and by printed leaflets was easy to access for large numbers of mental health patients and their primary caregivers at a relatively low cost. There has been an increasing interest recently demonstrating the effectiveness of psycho-education interventions using online, or booklets on patients and caregivers’ outcomes in relatively resource-poor countries [21].

Studies thus far have shown that these interventions may improve patients’ and primary caregivers’ outcomes, but the quality of included RCTs is poor due to a lack of randomization and blinding and insufficient sample sizes [22]. Consequently, the evidence base is inconclusive about the effectiveness of such interventions on patients and primary caregivers’ outcomes, hence the current study.

## Aim

The aim of this study is to investigate the the effectiveness of psycho-educational intervention delivered via a printed booklet on people diagnosed with Schizophrenia and their primary caregivers’ outcomes. It is anticipated that treatment as usual and psycho-educational intervention delivered by a booklet will: Improve patients’ and primary caregivers’ knowledge of schizophrenia, improve patients’ positive and negative symptoms and reduce their relapse rates, and improve primary caregivers’ burden of care and quality of life better than treatment as usual alone.

## Method/design

The study is a randomized controlled trial comparing the effect of psycho-educational intervention delivered via a printed booklet with treatment as usual on people diagnosed with Schizophrenia and their primary caregivers’ outcomes assessed at baseline, immediately post-intervention and at three months follow-up.

### Setting

The study will be conducted in four state funded psychiatric outpatient clinics in Amman, Jordan.

### Study participants

Participants will be people aged 18 years or over, diagnosed with schizophrenia or schizoaffective disorder, and their primary caregivers.

### Inclusion criteria

To be included in this study patients will have a primary diagnosis of schizophrenia according to the Diagnostic and Statistical Manual of Mental Disorders, 4^th^ Edition (DSM-IV) [23] and their primary caregivers. Caregivers are defined as the primary caregiver with close contact with the person. All participants should be able to read and write English or Arabic and be willing and able to consent.

### Exclusion criteria

Patients will be excluded if they have a learning disability, known organic mental disorder, have current substance abuse, living alone or without close contact with caregivers, or who are currently receiving any formal psycho-educational intervention. Primary caregivers will be excluded if they are involved in caring for more than one person with a diagnosed mental health problem.

### Recruitment

People diagnosed with schizophrenia or schizoaffective disorder in Jordan typically visits outpatient clinics with their family member. A poster about the study will be displayed in these clinics advertising the study and requesting volunteers. Interested participants will receive further information about the study directly from a research student or mental health professional in these clinics.

The study will recruit acute or chronic patients being treated in these clinics when they attend for appointments. AH or another mental health professional will inform participants about the study. The study information package will be given to each participant alongside a verbal explanation about the project. Sufficient time will be afforded to each participant to read the study information. Participants will be asked to return a signed consent form to nursing department in the clinic. AH will administer a study inclusion checklist to assess participants’ eligibility.

### Randomization

Participants who meet the inclusion criteria will be randomly allocated to one of the study arms by an independent research professional not connected to the study The allocation of participants to the study arm will be determined by a random number list generated by another researcher who has had no contact or access to recruited participants in the trial.

**Figure 1:**
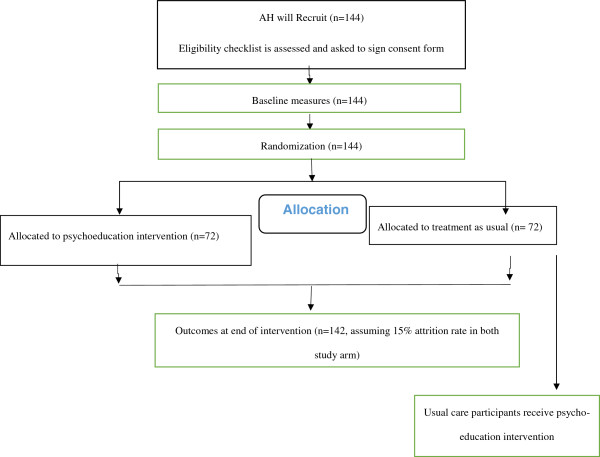
Flow diagram of study design.

Randomization will be implemented by using a third remote allocation system, PC will generate and send a random list to the independent researcher, AH will then contact the researcher when each participant is recruited, to receive the allocation. The allocation of participants to the study is shown in Figure 1.

## Intervention

### Psycho-education intervention

Besides treatment as usual, participants in the experimental arm of study will receive a psycho-educational booklets each fortnight for 12 weeks. Primary caregivers will receive follow-up phone calls to ensure that they have read and understood the booklet and to allow them to ask questions about its content. The psycho-educational intervention is based on the framework of Atkinson and Coia [24]. The booklets were developed by the research team and the content was checked, assessed and approved by an independent psychiatrist unconnected to the study. The booklets will be distributed to patients or caregivers during an outpatient visit. Booklets will be distributed inside a sealed envelope to maintain participants’ anonymity and minimize contamination between study arm participants. The booklet contents are shown in Table 1.

**Table 1:** Component of the psycho-educational intervention

**Booklet number**	**Contents**
One	-What is schizophrenia?-Common myths about schizophrenia.
	-Prevalence.
	-Disorder course.
	-Positive and negative symptoms of schizophrenia.
	-How is the schizophrenia diagnosed?
Two	-Stress vulnerability Model of psychiatric disorder.
	-Psychobiological vulnerability to disorders (genetic and biological factors).
	-Effects of alcohol and drug abuse and medication on symptoms.
	-How family members can help.
Three	-Medication effects on disorder (Treatment of acute symptoms).
	-The pros and cons of long term medication.
	-Strategies for managing side effects.
Four	-Early warning sign of relapse.
	-Definition.
	-Common early warning signs.
	-Identification of early warning signs for the client.
	-Explanation concept burden of care, importance of self-care and understanding the patients’ needs for help.
Five	-Recommendation for improving coexistence in the family.
	-Understand common difficulties.
	-Understand the concept of listening and empathy.
	-Problem solving strategies.
	-Managing disruptive behaviours.
Six	-Family expectation from patients and patients’ expectation from family.
	-Creating a low stress environment and ways to reduce stress.

### Treatment as usual (TAU)

TAU consists of medication, and laboratory investigations delivered by the mental health team.

### Measures

After recruitment and obtaining written consent from participants, but prior to randomization, baseline assessments will be taken by AH. Baseline assessment includes clinical information from the patient’s clinic record (medical diagnosis and illness duration) and demographic information; age, education level, employment status and marital status.

The primary outcomes for patients and primary caregivers are knowledge about schizophrenia measured by the Knowledge about Schizophrenia Questionnaire (KASQ). Secondary outcomes are schizophrenia symptoms measured by the Positive and Negative Symptom Scale (PANSS) for patients and Family Burden of Care measured by the Family Burden interview Scale (FBIS) and quality of life measured by the Schizophrenic Carers Quality of Life Scale (S-CQoL) for caregivers.

KASQ is a self-reported questionnaire developed by Ascher-Svanum [25]. It consists of 25 multiple-choice items intended to measure patients’ basic knowledge about schizophrenia and its management, aetiology, prevalence, prognosis, treatment and anti-psychotic medication effects and side effects. KASQ is scored from 0 to 25 with a higher score indicating more knowledge. KASQ has been used with people diagnosed with Schizophrenia and carers worldwide. Studies with subgroups [25] reported Cronbach’s alpha coefficients of 0.89 and 0.85 and test-retest reliability coefficient over three weeks was 0.83.

The PANSS was developed by Kay [26]. It assesses positive, negative and general psychopathology schizophrenia symptoms. The scale measures 30 clinical symptoms of schizophrenia, each symptom is scored from 1 indicating absence of psychopathology to 7 indicating severe psychopathology with higher scores indicating a poorer mental health status. The scale has good internal reliability and criterion related validity was examined with Andreasen rating systems. The result indicated that high correlation between two positive scales (r = 0.77, P < .0001) and negative scales (r = 0.77, P < .0001). And GPS from PANSS examined with Clinical Global Impression scale (CGI) and correlation was (r = 0.52, P < .0001) [27].

The FBIS was developed by Pai and Kapur [28] and assesses subjective, objective and global burden care of caring for mentally ill relatives at home. The FBIS consists of 24 items and focuses on six domains of caregivers’ burden: family finance, routine, leisure time, physical health, mental health and family interaction. Each item is rated on a three-point Likert scale (0: no burden, 1: moderate burden, 2: severe burden). The participants’ final scores range from 0 to 48; a higher score indicates a higher level of burden. The scale has good reliability and validity (Cronbach’s alpha 0.87; test-retest 0.83) [29].

The S-CQoL was developed by Richieri [30] to assess the impact of caring for people diagnosed with Schizophrenia on carers’ quality of life. The S-CQoL consists of 25 items scored from 25 to 125; a higher score indicates a good quality of life. The S-CQoL has seven dimensions: Physical and Psychological Wellbeing (PsPhW), Psychological Burden and Daily Life (PsBDL), Relationships with Spouse (RS), Relationships with Psychiatric Team (RPT), Relationship with Family (RFa), Relationships with friends (RFr) and Material Burden (MB). The Scale has an adequate internal consistency for all dimensions ranging from 0.64 to 0.95 and Cronbach’s alpha is 0.79 to 0.92. External validity examination was shown a significantly correlated S-QoL dimension with all short form-36 dimension scores (SF-36) [30].

In reviewed literature, there were disparities in the operational definition of relapse. However, the majority of these studies adopted numbers of readmission, or exacerbations in psychotic symptoms of more than 5–10 points measured on the Brief Psychiatric Rating Scale (BPRS) or PANSS, the number of increases in anti-psychotic medication dosage, or medication compliance. For this study, relapse is defined as the number of readmissions measured at baseline three months prior to the study commencing, at end of intervention immediately and three months follow up. Furthermore, relapse with medication means the numbers of increasing current antipsychotic medications prescribed to patients during the same intervals. These data will be obtained from participants’ medical records.

None of these outcomes have been used in an Arabic speaking country so they will be translated from English to Arabic and back translated to English, checked for any discrepancies by independent bilingual translator and original author for each instrument to maintain content validity. Prior to the main study, we will recruit two patients and two carers to test participants’ acceptability and understanding of the scales.

All measures will be assessed at baseline, immediately post-intervention and at 3 month follow-up.

### Blinding

Participants will be aware of which group, to which they have been allocated. In addition, the researchers are also aware of participants’ allocation. In order to minimize potential bias AH will assess baseline measures prior to allocation; post intervention and follow-up measures will be assessed by an independent researcher unaware of the participants’ allocation.

### Sample size

We are seeking to detect a mean KASQ improvement two scores (8%) in the intervention arm and have estimated that with a power of 80% and significance level of p < 0.05, allowing for 15% attrition, deduced from previous studies, 144 participants will be required.

### Data analysis

The first stage of the quantitative data analysis will describe participants’ responses to the continuous variables, i.e. KASQ, PANSS, FBIS and S-CQOL scores and patients’ total and mean relapse rates. The next stage of the data analysis will compare KASQ, PANSS, FBIS and S-CQOL scores, and patient relapse rates between the intervention and control arms using independent sample t tests, or non-parametric equivalents if the data are not normally distributed. Effect sizes, 95% confidence intervals (CI), Numbers needed to treat (NNT) and odds ratios will be reported. The third stage of the data analysis will use regression analyses to identify which of the baseline measures are statistically significant predictors of post intervention and follow-up outcomes. The final stage of the data analysis will investigate pre and post intervention differences on all measures *within* the intervention and control arms using a related t test or non-parametric equivalent if the data are not normally distributed. The level of significance for all analyses will be set at p < 0.05. Analysis will be conducted using the Statistical Package for the Social Sciences (SPSS) version 21.

### Ethical approval

Ethical approval has been obtained from University of Nottingham Faculty of Medicine and Health Sciences Research Ethics Committee (SNMP 12072012) and the Scientific Research Ethics Committee from Ministry of Health, Jordan (Ref 9067). The trial has been registered with Current Controlled Trials, registration number ISRCTN78084871.

## Discussion

Findings from this trial will show the effect of psycho-education delivered via printed booklets, and treatment as usual on outcomes for people diagnosed with Schizophrenia and their primary caregivers when compared with treatment as usual alone.

## Trial status

The trial commenced recruitment in September 2012.

## Abbreviations

RCT: Randomized controlled trial; MoH: Ministry of health; KASQ: Knowledge about schizophrenia questionnaires; PANSS: Positive and negative syndrome scale; FBIS: Family burden interview schedule; S-CQoL: Schizophrenia –caregivers’ quality of life; CGI: Clinical global impression scale; GPS: Clinical global impression scale; BRPS: Brief psychiatric rating scale; NNT: Number need to treat; CI: Confidence interval; SNMP: School of nursing & midwifery; AH: Abd Hasan; PC: Patrick Callaghan.

## Competing interests

The authors declare that they have no competing interests.

## Authors’ contributions

AH, PC and JL were responsible for the development and refinement of the protocol. AH is the principle investigator. AH will conduct data analysis under the supervision of PC & JL. AH, PC and JL wrote the initial draft of the manuscript and contributed to, edited and approved the final manuscript.

## Pre-publication history

The pre-publication history for this paper can be accessed here:

http://www.biomedcentral.com/1471-244X/14/17/prepub
